# Xiaoqinglong Decoction Attenuates Chronic Obstructive Pulmonary Disease in Rats via Inhibition of Autophagy

**DOI:** 10.1155/2018/6705871

**Published:** 2018-01-31

**Authors:** Huanan Wang, Bing Mao, Chang Chen

**Affiliations:** ^1^Department of Integrated Traditional Chinese and Western Medicine, West China Hospital of Sichuan University, Chengdu 610041, China; ^2^Institute of Life Sciences, Chongqing Medical University, Chongqing 400016, China

## Abstract

Effective treatment for chronic obstructive pulmonary disease (COPD) and knowledge of the underlying mechanism are urgently required. Xiaoqinglong decoction (XQL) is widely used to treat COPD in Traditional Chinese Medicine, but the mechanism remains unclear. In this study, we tested the hypothesis that XQL ameliorates COPD via inhibition of autophagy in lung tissue on a rat model. Rats were divided into five groups, namely, Control group, COPD group, COPD + XQL group, COPD + Rapamycin group, and COPD + XQL + Rapamycin group. Pathological changes on cellular and molecular levels, apoptosis reflected by TdT-mediated dUTP Nick-End Labeling (TUNEL) assay, and autophagy represented by LC3II/LC3I ratio and p62 level were investigated for each group. Compared with the Control group, COPD rats exhibited structural changes and activated inflammation in the lung tissue, together with enhanced apoptosis and elevated autophagy biomarkers. XQL treatment significantly ameliorated these changes, while rapamycin augmented them. These data altogether confirmed the involvement of autophagy in the pathogenesis of COPD and suggested that XQL attenuates COPD via inhibition of autophagy.

## 1. Introduction

COPD is an obstructive, long-term lung disease characterized by airway remodeling, chronic airway inflammation, and excessive mucus secretion [1]. It ranked the 5th cause of death as of 2002 [2] and occurred in 2.4% of the global population as of 2015 [3]. However, no effective treatment for COPD is available. Hence, it is urgently required to understand its pathogenesis and discover new therapies [4]. 

Smoking is the major cause of COPD [5]. Long-term exposure to smoke causes inflammation, which consequently narrows down the small airways and breaks lung tissue [6]. The inflammation is associated with activation of the transcription factor nuclear factor-*κ*B (NF-*κ*B) by tumor necrosis factor- (TNF-) *α*, which induces IL-8 secretion in airway cells [4]. TNF-*α* and IL-8 concentrations in sputum are closely associated with the severity of the disease [4]. Rat exposed to cigarette smoking is a widely applied animal model of COPD [1]. Typical COPD indicators used to verify the success of the model include structural changes such as bronchiole and arteriole wall thickness and airway narrowing, lung function changes such as airway resistance, inflammation indicators such as inflammatory cells and mediators (TNF-*α*, IL-8, etc.), and apoptosis [1].

Autophagy is a mechanism that cell disassembles unnecessary or dysfunctional cytoplasmic material of endogenous or exogenous origin within lysosomes [7]. This mechanism has profound implications for a number of pathophysiological processes in nervous, cardiovascular, immune, and pulmonary systems, as well as in aging [7]. Autophagy can be triggered by environmental factors; thus it plays an important part of adaptive responses in stress [8].

The involvement of autophagy in lung diseases was hypothesized in 2000 when autophagic vacuoles were observed in the liver tissue of a patient with *α*1AT-deficiency, a mutation associated with the development of emphysema [9]. This hypothesis was supported by subsequent studies showing that the expression of LC3B-II, the key autophagy-associated protein, and autophagosome formation were increased in COPD lungs as compared with controls. [10, 11]

Xiaoqinglong decoction (XQL), which was recorded in Treatise on Febrile Diseases in Han Dynasty of China, is used to treat diseases of respiratory system including cough, asthma, and COPD [12]. It is prepared from eight herbal materials, namely, ephedra herb, Radix Paeoniae Alba,* Asarum sieboldii* Mig., Rhizoma Zingiberis, honey-fried licorice root, cassia twig,* Schisandra chinensis*, and* Pinellia ternata*. Although the effectiveness of XQL on COPD has been verified in both clinical trials and animal models, the mechanism remains unclear, which limits its further applications [12].

In this study, we hypothesized that XQL exhibited its anti-COPD effect via suppression of autophagy in lung tissue. To test the hypothesis, we firstly observed the autophagy indicators together with COPD symptoms in smoke-induced COPD rat model to confirm if the animal model was successful and whether autophagy was involved in COPD. Secondly, we examined the changes of the abovementioned index when these animals were given XQL and the classic autophagy inducer rapamycin, respectively, or simultaneously, to demonstrate the effectiveness of XQL in attenuating COPD and its association with autophagy.

## 2. Materials and Methods

### 2.1. XQL Preparation

XQL was prepared based on the traditional method. Specifically, 14 g of ephedra herb, 16 g of cassia twig, 14 g of Radix Paeoniae Alba, 18 g of* Schisandra chinensis*, 18 g of Rhizoma Zingiberis, 54 g of* Pinellia ternata*, 36 g of honey-fried licorice root, and 18 g of* Asarum sieboldii* Mig. were first soaked in 1600 mL of water for 30 min. Then the mixture was boiled until the total volume dropped to 500 mL, which took approximately another 30 min. The decoction was filtered with gauze, subaliquoted into fourteen 50 mL plastic tubes, and stored at −20°C. On each experimental day, one tube was defrosted at room temperature prior to XQL administration.

### 2.2. Animals and Treatments

Thirty SD rats aged 6−8 weeks and weighed 230−250 g were equally randomized into five groups (*n* = 6 in each group): Control group, which underwent no treatment; COPD group, which were COPD rats without further treatment; COPD + XQL group, which were COPD rats treated with XQL; COPD + Rapamycin group, which were COPD rats given rapamycin; and COPD + XQL + Rapamycin group, which were COPD rats treated with XQL and rapamycin. Comparisons between the Control and the COPD groups showed whether the COPD model was successful and whether autophagy was activated in COPD. Comparisons between the COPD group and the COPD + XQL group aimed to reveal the effectiveness of XQL and if this effect was autophagy-associated; comparisons between the COPD + Rapamycin group and the COPD + XQL + Rapamycin group further shows whether XQL counteracts with the activation of autophagy induced by rapamycin.

All rats were kept in a temperature-controlled room (21°C) in 12 h/12 h light/dark cycles and had free access to tap water and normal chow. The handling and treatment of animals were performed in accordance with the guidelines of the Experimental Animal Care and Institutional Animal Ethical Committee of West China Hospital of Sichuan University.

COPD model was established via combinational use of cigarette smoke, lipopolysaccharide (LPS), and cold stimuli. Specifically, on day 1 and day 14, 200 *μ*g/kg body weight of LPS (prepared as 20 *μ*g/mL solution in saline) was delivered to the throat of the rats using a plastic tube after anesthesia with diethyl ether, followed by keeping the rats in upright position for 15 seconds to facilitate the distribution of LPS in the lungs. On each day of days 2−13 and days 15−28, the rats were kept in a covered box (70 cm × 60 cm × 60 cm) and exposed to the smoke from fifteen continuously burned cigarettes. Each cigarette was burned out within 3 minutes. The smoke concentration was calculated to be 100−120 mg/m^3^ for a period of 45 minutes. After the smoke exposure, the rats were immediately transferred into a room at 16°C and kept there for 1 hour.

From day 29 to day 43, 2 mL of XQL was orally administered to each rat in the COPD + XQL group and the COPD + XQL + Rapamycin group. Rats in the Control group, the COPD group, and the COPD + Rapamycin group received equivalent volume of water by gavage. In addition, the COPD + Rapamycin group and the COPD + XQL + Rapamycin group were intraperitoneally injected with 0.2 mg/kg of rapamycin, while rats in the Control, COPD, and COPD + XQL groups were injected with 0.2 mL of saline. Body weights of all rats were recorded daily. On day 44, blood samples were collected from the rats' hearts after anesthesia. After the rats stopped breathing, the lungs were collected, perfused with cold (4°C) saline, transferred into liquid nitrogen immediately, and stored at −80°C until analysis. Serum was obtained by centrifuging coagulated blood samples at 3000 g and 4°C for 5 min.

### 2.3. HE Staining

The lower right lung tissue was fixed by 4% paraformaldehyde for 24 h, dehydrated in alcohol for 12 h, embedded in paraffin, cut into 4 *μ*m sections, stained with hematoxylin and eosin, and mounted onto a glass slide. The sections were observed and photographed under a light microscope by a pathologist without knowledge of their origins.

The mean linear intercept (MLI), an indicator of alveolar size, and mean alveolar number (MAN), an indicator for density of alveoli, were determined to assess the degree of lung emphysema. The number of linings (NS) and the total alveolar count (Na) were calculated, and the area of the image (S) was measured in a HE photograph. The MLI and MAN were calculated according to the formula MLI = L/NS and MAN = Na/S. For each indicator, the average value was calculated from six randomly selected fields, each from the lung of one rat [13].

### 2.4. TdT-Mediated dUTP Nick-End Labeling (TUNEL)

Apoptosis of the lung was evaluated using TUNEL assay with TUNEL Apoptosis Assay Kit E607172 purchased from Sangon Biotech (Shanghai) Co., Ltd. (Shanghai, China), following the manufacturer's instructions. Briefly, the paraffin sections described above were deparaffinized with xylene and alcohol, permeabilized with proteinase K at 37°C for 15 min, and incubated consecutively with TDT enzyme solution and antibodies. The processed sections were observed and photographed under a CKX41SF fluorescence microscope (Olympus Corporation, Tokyo, Japan).

### 2.5. ELISA

Serum levels of the inflammation indicators IL-8 and TNF-*α* were measured using ELISA kits (NeoBioscience, Shenzhen, China) following the manufacturer's instructions. A Multiskan MK-3 microplate reader (Thermo Fisher Scientific, Finland) was employed to read the absorbance.

### 2.6. Western Blot Analysis

Western blotting was applied to quantify LC3 and p62 proteins in the lung tissue. The left lung was weighed and protein extracted. Approximately 5 mg of tissue was homogenized and centrifuged at 12000*g* and 4°C for 10 min. The supernatant was transferred into another vial and the protein concentration determined using BCA protein assay kit (Beyotime® Biotechnology, Shanghai, China). The protein samples were suspended in 5 × loading buffer, denatured for 5 min at 100°C, separated by 15% sodium dodecyl sulfate-polyacrylamide gel electrophoresis (SDS-PAGE), and transferred onto nitrocellulose membranes by electroelution (200 mA). The blots were blocked with 5% nonfat milk in Tris-buffered saline containing 0.1% Tween-20 (TBST) at 37°C for 2 h and then incubated overnight with primary antibodies against LC3 (1 : 500; Proteintech), p62 (1 : 1000; Affinity), and *β*-actin (1 : 2000; Abcam) at 4°C. Then the membranes were washed with TBST and exposed to secondary antibody (1 : 3000) for 1.5 h at room temperature. After three washes with TBST, immunoreactive proteins were visualized using the ChemiDoc XRS + System (Bio-Rad Laboratories, Inc., USA). The densitometry values were expressed as the targeted protein/*β*-actin ratio.

### 2.7. Statistical Analysis

All statistics were based on the six animals in each group. Data were expressed as mean ± SD and compared using one-way ANOVA and following Least Significant Difference test by SPSS 16.0 (IBM Corporation, New York). *p* < 0.05 was considered significant.

## 3. Results and Discussion

### 3.1. Pathological Changes

**Figure 1:**
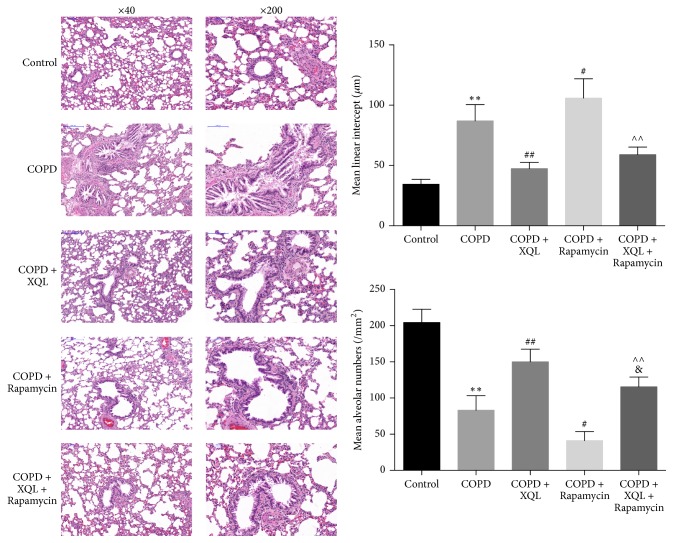
HE staining of lung tissue. The bar graphs show semiquantification of mean linear intercept (MLI) and mean alveolar number (MAN). ^*∗∗*^*p* < 0.01 versus Control; ^#^*p* < 0.05, ^##^*p* < 0.01 versus COPD; ^&^*p* < 0.05 versus COPD + XQL; ^∧∧^*p* < 0.01 versus COPD + Rapamycin, *n* = 6; data were presented as mean ± SD.

Compared with the Control rats, the COPD rats displayed changes in microstructure of bronchus, namely, narrowed airways and airway wall inflammation, the latter featured by increased infiltration of inflammatory cells in the airway walls. XQL administration partially reversed these changes, as shown in the COPD + XQL group. Rapamycin administration in the COPD + Rapamycin and COPD + XQL + Rapamycin groups worsened the pathological changes as compared with the COPD and COPD + XQL groups, respectively. The comparison between the COPD + Rapamycin and COPD + XQL + Rapamycin group showed that the latter experienced less structural changes than the former (Figure 1).

Semiquantitative measurements showed that MLI increased significantly in the COPD rats. Rapamycin augmented the change while XQL attenuated it. Likewise, MAN decreased significantly in the COPD rats. This trend was amplified by rapamycin but inhibited by XQL (Figure 1).

**Figure 2:**
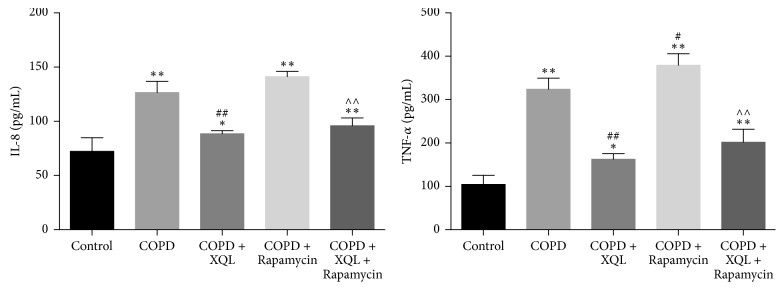
Serum IL-8 and TNF-*α* levels in each group. ^*∗*^*p* < 0.05, ^*∗∗*^*p* < 0.01 versus Control group; ^#^*p* < 0.05, ^##^*p* < 0.01 versus COPD group; ^∧∧^*p* < 0.01 versus COPD + Rapamycin group, *n* = 6; data were presented as mean ± SD.

ELISA results (Figure 2) showed that the COPD rats exhibited higher serum levels of IL-8 and TNF-*α* than the controls. XQL treatment (COPD + XQL group) significantly attenuated these changes while rapamycin treatment (COPD + Rapamycin group) significantly enlarged the TNF-*α* increase. Compared with the COPD + Rapamycin group, the COPD + XQL + Rapamycin group displayed significantly higher levels of IL-8 and TNF-*α*, suggesting that rapamycin enhanced the COPD inflammation, while XQL attenuated it.

Body weight of each rat was recorded throughout the study (Supplementary Materials, Table S1). Generally, the average body weight of the Control rats was higher than all the other groups, suggesting the harm of COPD on the animals' health. Compared with the COPD + Rapamycin rats, the COPD + XQL + Rapamycin rats showed higher body weight, indicating a protective effect of XQL on the rats' general wellbeing.

### 3.2. Apoptosis

**Figure 3:**
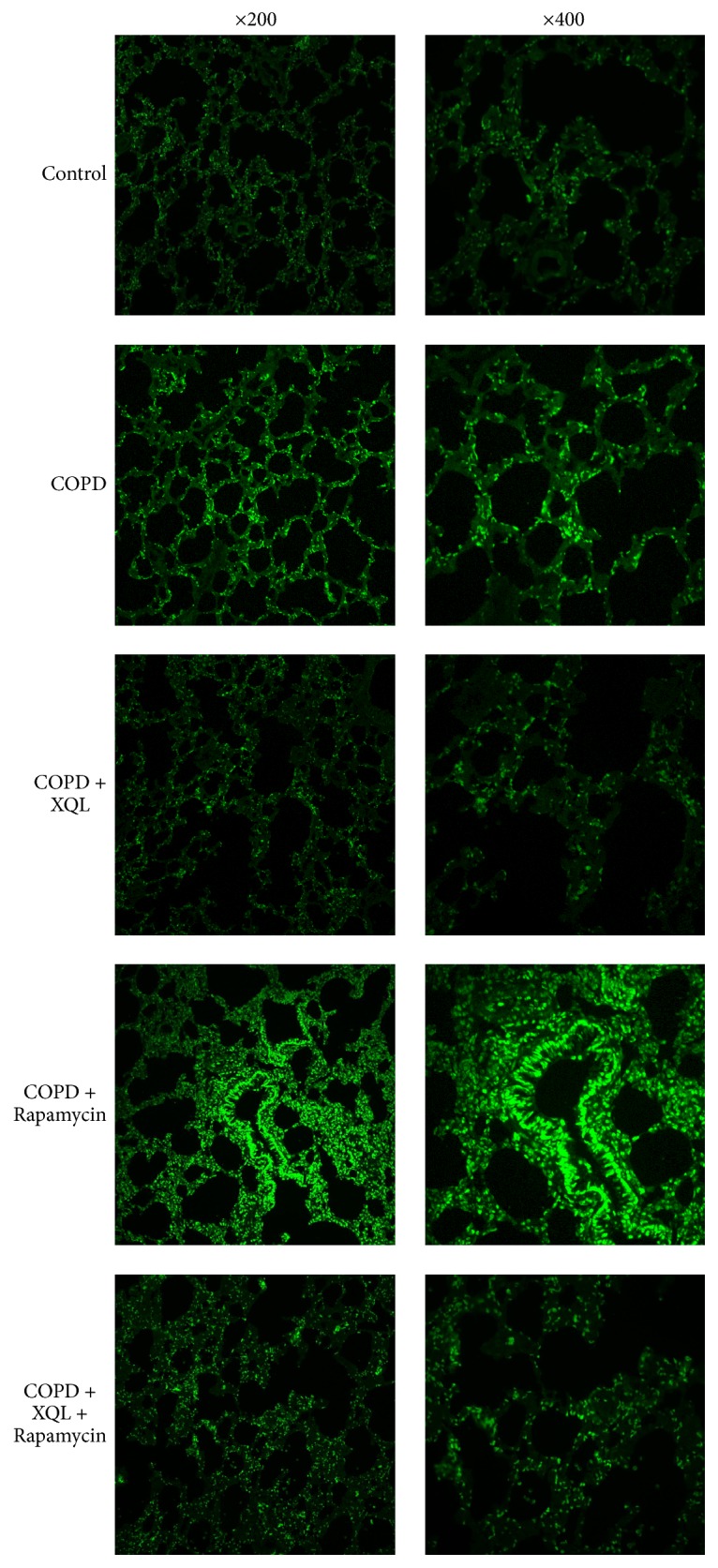
TUNEL results of lung tissue. Intensity of fluorescence reflects the apoptosis level.

TUNEL test revealed that, compared with the Control group, the rats in COPD group exhibited higher levels of apoptosis (Figure 3). Compared with the COPD group, the apoptosis level of rats in the COPD + XQL group was lower, while that of the COPD + Rapamycin group was higher. The COPD + XQL + Rapamycin group displayed lower apoptosis activity than the COPD + Rapamycin group but higher activity than the COPD + XQL group. The above data shows that rapamycin amplified apoptosis in the COPD rats, while XQL administration counteracted this effect.

### 3.3. Autophagy

**Figure 4:**
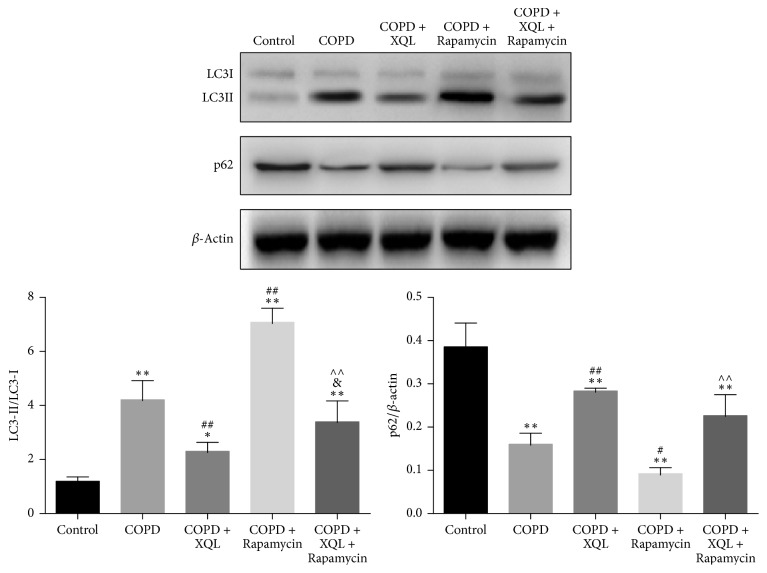
Western blotting of LC3I, LC3II, p62, and *β*-actin. Densitometry values were expressed as the targeted protein/*β*-actin ratio. ^*∗*^*p* < 0.05, ^*∗∗*^*p* < 0.01 versus Control group; ^#^*p* < 0.05, ^##^*p* < 0.01 versus COPD group; ^&^*p* < 0.05 versus COPD + XQL group; ^∧∧^*p* < 0.01 versus COPD + Rapamycin group, *n* = 6; data were presented as mean ± SD.

Figure 4 showed western blotting results of LC3II/LC3I ratio and p62 levels in each group. The COPD rats, with and without further treatments, displayed higher LC3II/LC3I ratios. Compared with the COPD rats, the COPD + XQL group showed lower LC3II/LC3I ratios, while the COPD + Rapamycin group showed higher LC3II/LC3I ratios. The COPD + XQL + Rapamycin group exhibited higher ratio of LC3II/LC3I than the COPD + XQL group, but lower LC3II/LC3I ratio than the COPD + Rapamycin group.

In regard to p62, the levels in all four COPD groups were significantly lower than the Control group. The COPD + XQL group showed higher p62 levels than the COPD group, while the COPD + Rapamycin group showed lower p62 levels than the COPD group. Besides, the COPD + XQL + Rapamycin group displayed higher p62 levels than the COPD + Rapamycin group.

These results indicated that the LC3II/LC3I ratio was upregulated and p62 level downregulated in COPD rats and that XQL could attenuate or reverse these changes. The protective effects induced by XQL were counteracted by rapamycin, the autophagy activator, suggesting opposite effects of XQL and rapamycin.

The COPD mice in this study exhibited typical symptoms and characteristics of the disease. XQL treatment ameliorated these symptoms, while rapamycin worsened them. The autophagy biomarker, LC3II/LC3I ratio, was elevated while p62 concentration was decreased in the COPD rats; these changes were enlarged by rapamycin administration but attenuated by XQL treatment. These data altogether exhibited the effect of XQL, which was opposite to the autophagy activator rapamycin.

Rapamycin activates autophagy via inhibition of the mammalian target of rapamycin mTOR [14]. The process is accompanied by decreased expression of p62 and increased expression of LC3 [15]. When autophagosome is formed, LC3-I is processed to LC3-II by lipidation; hence the ratio of LC3-II/LC3-I is a marker of autophagy [16]. Both phenomena were observed in this study (Figure 4).

Involvement of autophagy in COPD has been confirmed by a number of studies. For example, Chen et al. found that microtubule-associated protein 1 light chain 3 beta (LC3B) activated apoptosis in the cigarette smoke-induced emphysema mice model, suggesting autophagy is responsible for the apoptosis in lung diseases [17].

Herbal medicine has been used to treat lung diseases for a long time. Apart from XQL, other herbal medicines, such as* Alisma orientale,* are also prescribed as a remedy to lung diseases such as edema and COPD. A study on the mechanism of ethanol extract of the tuber of* Alisma orientale* on COPD on a cellular model revealed that this effect was due to suppression of LC3II, which was associated with activation of mTOR [18]. Similar phenomenon was also observed in the present study, which showed suppression of autophagy by XQL.

Hence, it raises a question of which component in XQL is responsible for its effect on COPD and autophagy. XQL is traditional Chinese medication prepared from eight herbs, among which only one plant,* Schisandra chinensis*, was investigated for its effect on autophagy so far. However, ingredients in* Schisandra chinensis* were found to activate, rather than inhibit, autophagy in these studies. For example, Schisandrin A, a component in* Schisandra chinensis*, displays liver protective effect via activation of autophagy flux and inhibition of apoptosis in mice [19]. Schisandrin B, an antioxidant lignan from* Schisandra chinensis*, protected neurons via activation of autophagy in A*β*-infused rats [20].

A summary of the abovementioned studies on herbal medicine and autophagy shows that all of the herbal medicine exhibited protective effect. However, XQL and* Alisma orientale* protected lung tissue against COPD by inhibiting autophagy, while* Schisandra chinensis, *one component in XQL, exhibited its protective effect against liver and neuron damage via activation of autophagy. These phenomena look contradictory but may actually be consistent, because although activation of autophagy exhibits protective effect in many diseases, it plays a deleterious role in COPD. This may be due to the fact that prolonged exposure of smoke continuously activates autophagy and exceeds the beneficial aspect of autophagy-mediated cell death [8]. Hence, the protective effect of herbal medicine on COPD may be due to their inhibition of autophagy, while the protective effect on other tissue damage is due to the activation of autophagy.

It should be noted that, considering that XQL is a combination of eight herbs and each one contains a variety of components, the ingredients and compounds responsible for the pharmacological effects observed in this study remain unclear, which is a challenge in all studies on herbal medicine.

Another limitation of the study is that no sample was collected between completion of modeling and commencement of XQL treatment. Thus, it was unclear whether XQL prevented or reversed the progress of COPD. Nevertheless, the study clearly shows that XQL possesses protective effect on COPD in rats, which is associated with the inhibition of autophagy.

## 4. Conclusions

To summarize, this study supports the hypothesis that XQL inhibits the progress of COPD via attenuating the autophagy process. Further studies are required to investigate the key components in the decoction that are responsible for this effect.

## Supplementary Materials

Supplementary Materials Table S1: the body weights of the rats in each group and week, which indicates general wellbeing of the rats. Body weights (grams) of the rats in each group and week of treatment.
