# Supplementary material for: Xiaoqinglong Decoction Attenuates Chronic Obstructive Pulmonary Disease in Rats via Inhibition of Autophagy
Source: Evid Based Complement Alternat Med. 2018 Jan 31;2018:6705871. doi: 10.1155/2018/6705871 (PMC5831972; doi:10.1155/2018/6705871)
Supplement: Supplementary Materials — Table S1: the body weights of the rats in each group and week, which indicates general wellbeing of the rats. Body weights (grams) of the rats in each group and week of treatment. [file 6705871.f1.docx]

Table S1. Body weight (gram) of the rats in each week of treatment

|  | Control | COPD | COPD+XQL | COPD+Rapamycin | COPD+XQL+Rapamycin |
| --- | --- | --- | --- | --- | --- |
| Week 0 | 237.00±1.73 | 234.67±4.04 | 233.67±4.04 | 240.00±2.65 | 243.00±1.73 |
| Week 1 | 287.33±9.02 | 252.00±10.00** | 243.67±6.51** | 256.67±13.80** | 277.33±9.29^&&^^ |
| Week 2 | 347.67±15.95 | 303.67±15.95** | 291.00±4.58** | 302.00±20.88** | 331.00±10.82^&&^^ |
| Week 3 | 370.67±22.37 | 297.67±3.79** | 301.00±24.00** | 301.67±8.14** | 328.33±13.20** |
| Week 4 | 394.67±27.47 | 334.33±13.87** | 319.67±3.79** | 328.67±29.02** | 362.33±13.58^&^ |
| Week 5 | 422.67±30.02 | 357.67±19.43** | 336.00±4.36** | 327.00±27.00** | 365.33±9.50**^ |
| Week 6 | 446.67±29.19 | 386.00±19.67** | 348.00±3.46**^##^ | 334.67±24.54** | 366.67±7.02** |

Data are expressed as mean ± SD, n = 6 for each group. **p<0.01 vs. Control；^##^p<0.01 vs. COPD group；^&&^p<0.01 vs. COPD+XQL group；^p<0.05 vs. COPD+Rapamycin group.
